# Sward type alters the relative abundance of members of the rumen microbial ecosystem in dairy cows

**DOI:** 10.1038/s41598-020-66028-3

**Published:** 2020-06-09

**Authors:** Paul E. Smith, Daniel Enriquez-Hidalgo, Deirdre Hennessy, Matthew S. McCabe, David A. Kenny, Alan K. Kelly, Sinéad M. Waters

**Affiliations:** 1 Teagasc, Animal and Bioscience Research Department, Animal & Grassland Research and Innovation Centre, Teagasc, Grange, Dunsany Ireland; 2 UCD College of Health and Agricultural Sciences, University College Dublin, Belfield, Ireland; 3 Teagasc, Animal & Grassland Research and Innovation Centre, Moorepark, Fermoy, County Cork Ireland; 4 University of Bristol, Bristol Veterinary School, Langford, Somerset, BS40 5DU UK; 5 Departamento de Ciencias Animales, Facultad de Agronomía e Ingeniería Forestal, Pontificia Universidad Católica de Chile, Santiago, Chile

**Keywords:** Applied microbiology, Microbiome

## Abstract

The performance of ruminant livestock has been shown to benefit from the enhanced nutritive value and herbage yield associated with clover incorporation in the grazing sward. However, little research to date has been conducted investigating the effects of mixed swards containing white clover on the composition of the rumen microbiome. In this study, the rumen microbial composition of late lactation dairy cows grazing perennial ryegrass only (PRG; n = 20) or perennial ryegrass and white clover (WCPRG; n = 19) swards, was characterised using 16S rRNA amplicon sequencing. PERMANOVA analysis indicated diet significantly altered the composition of the rumen microbiome (P = 0.024). Subtle shifts in the relative abundance of 14 bacterial genera were apparent between diets, including an increased relative abundance of *Lachnospira* (0.04 vs. 0.23%) and *Pseudobutyrivibrio* (1.38 vs. 0.81%) in the WCPRG and PRG groups, respectively. The composition of the archaeal community was altered between dietary groups, with a minor increase in the relative abundance of *Methanosphaera* in the WCPRG observed. Results from this study highlight the potential for sward type to influence the composition of the rumen microbial community.

## Introduction

Forage is recognised as the cheapest source of nutrition for ruminant livestock^1^ and predominates as the main source of feed in temperate climatic regions such as Ireland^2^. The addition of white clover (*Trifolium repens*) in the grazing sward has been reported to increase herbage yield^3–5^ and enhance animal performance in both dairy and sheep systems^6–10^. However the positive response to milk production associated with clover inclusion at pasture can be varied^11,12^. Legumes tend to have increased protein content and a reduced proportion of fibre in comparison to grasses^13^. Indeed, it is these attributes that are common to legumes, which has resulted in the majority of authors concluding improvements to animal performance to arise from increased feed intakes (due to reduced NDF) and overall increase to the sward nutritive value (as a result of increased CP) associated with clover inclusion in pastoral systems^4,7,10^.

A reduction to the fibrous proportion of diets is associated with increased feed consumption in ruminants^14^. The decreased fibre proportion in legumes compared to grasses^13^ most likely results from a combination of the increased pectin^15,16^ and reduced hemicellulose content in clover^17^. Differences in the carbohydrate composition between both forages results in a contrasted microbial degradation when ingested. The degradation of the pectin component of the cell wall in legumes^18^ results in a more rapid rate of degradability in the rumen^19^ most likely explaining the associated increase in feed intake when clover is fed^20^, due to a swift reduction in particle size.

The regulatory capacity of diet on the composition and functionality of the rumen microbiome has long been recognised resulting from the effects of a varied supply of growth substrates for differing microbes^21–23^. Subsequently, differences in the composition of the rumen microbial communities are known to influence animal performance^24–26^. Therefore, all dietary strategies under investigation and/or currently utilised on farm, should consider animal performance in tandem with rumen microbial composition and function.

To the best of our knowledge, only two studies have evaluated the effects of grazing swards containing white clover on the composition of the rumen microbiome using next generation sequencing techniques^27,28^. Both studies reported minor or no effects on the rumen microbial composition, although the potential for white clover inclusion in the sward to cause changes in the rumen microbiota has been suggested^27^.

Monocultures of white clover were investigated by Bowen *et al*.^27^, which would not regularly be grazed at farm level. As a result, the effect of white clover feeding, under practical on farm conditions, on the rumen microbiome, has only been conducted in one study and requires further validation. In addition, the grazing of clover swards has been reported to have a variable effect on methane production^12,20,29,30^. However, no simultaneous data on the composition of the rumen microbiome and methane production of ruminants grazing clover swards is available.

Therefore the aim of this study was to investigate if the inclusion of white clover in the grazing sward (WCPRG) would alter the composition and predicted functionality of the rumen bacteria and archaea communities of dairy cows, in comparison to monocultures of perennial ryegrass (PRG). Rumen microbial samples were obtained from a previous experiment investigating the effects of sward type on animal performance and enteric methane production^12^. An additional aspect of this study was to determine if minor differences in methane yield associated with sward type, would be reflective of alterations to the rumen archaeal population.

## Results

Animal performance and herbage analysis has previously been reported by Enriquez-Hidalgo *et al*.^12^.

### DNA Extraction and Sequencing Run Performance

No evidence of DNA degradation associated with the long term storage of samples was present with agarose gels revealing bands predominantly smeared between 12 kb and 1 kb positions for all samples. This range of smearing is to be expected due to DNA shearing associated with the bead beating method utilised^31^.

A total of 13,513,572 reads were obtained from sequencing on the Illumina MiSeq with an average 168,307 ± 36,207 reads per sample. Following quality filtering, merging and removal of chimeric sequences, there was a total of 5,543,164 reads with 138,579 ± 32,416 reads per sample.

A correlation of (r_s_ = 0.976) was observed between the theoretical composition of the ZymoBIOMICS^TM^ DS standard and that of our generated DS library. Run performance was deemed to have been performed to an optimal standard based on the correlation between our internal positive control and the theoretical composition supplied by ZymoBIOMICS^TM^.

### Microbial Community Structure and Composition

No effect of sward type was observed on *alpha* diversity in the rumen microbial community structure, with no difference detected in Shannon or Simpson metrics (P = 0.23 and P = 0.34), respectively. Due to poor species classification, all ASVs within each sample were classified to the genus level. This resulted in a total of 304 taxa being identified at the genus level across all samples.

Across both diets, bacteria and archaea had an average relative abundance of 98.32 and 1.68%, respectively. At the phylum level, *Bacteroidetes* and *Firmicutes* dominated, with *Prevotellaceae*, *Lachnospiraceae* and *Ruminococcaceae* residing as the most abundant family of taxa across all samples. Finally, *Prevotella 1* (40.58%) was the most abundant taxa identified in all samples when classified at the genus level. Other abundant genera of microbes observed across all samples included *Christensenellaceae R-7 group* (5.59%), *Rikenellaceae RC9 gut group* (4.81%), *Fibrobacter* (4.69%) and *Ruminococcaceae NK4A214 group* (3.93%).

### Dietary Effect on Rumen Microbial Community

**Figure 1:**
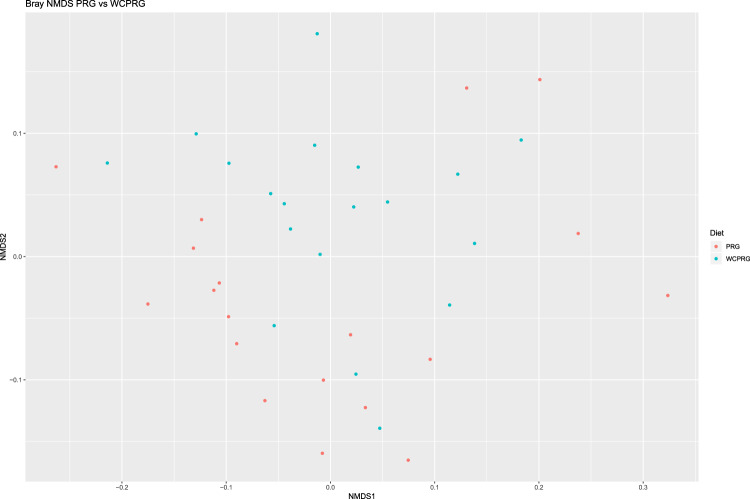
Bray-Curtis NMDS plot highlighting differences in the bacterial and archaeal community composition between treatments. Different colour dots represent samples obtained from animals grazing different swards. Red = Perennial ryegrass (PRG), Blue = Perennial ryegrass and white clover (WCPRG).

A NMDS plot of overall microbial community sample data, generated using Bray-Curtis dissimilarity analysis (Fig. 1), displayed a moderate degree of clustering of samples based on diet with respect to the microbial communities. Community composition was deemed to have been altered by diet based on the results of the PERMANOVA analysis (P = 0.024).

*Cyanobacteria* were the only phyla with a relative abundance that was significantly altered by diet, with an increased abundance noted in the PRG group (Table 1). Dietary composition resulted in a significant increase in the abundance of *Veillonellaceae* in the PRG group (Table 2). Additionally, a minor but significant, rise in the abundance of *Desulfovibrionaceae* was observed in the PRG group.

**Table 1:** Mean relative abundance (%) of the ten most abundant phylum in each dietary group.

Kingdom	Phylum	WCPRG	PRG	*P*-value	adj *P*- value
Bacteria	Bacteroidetes	48.63%	47.53%	0.58	0.73
Bacteria	Firmicutes	31.97%	31.41%	0.64	0.77
Bacteria	Kiritimatiellaeota	5.25%	6.66%	0.09	0.24
Bacteria	Fibrobacteres	3.74%	3.42%	0.22	0.43
Bacteria	Tenericutes	2.66%	2.24%	0.10	0.26
Bacteria	Cyanobacteria	1.27%	2.10%	0.00	<0.05
Bacteria	Spirochaetes	1.50%	1.51%	0.77	0.88
Archaea	Euryarchaeota	1.29%	1.58%	0.03	0.15
Bacteria	Patescibacteria	1.33%	1.11%	0.02	0.15
Bacteria	Proteobacteria	1.17%	1.17%	0.89	0.96

PRG = Perennial ryegrass only; WCPRG = Perennial ryegrass and white clover sward;

*P*- values were generated using the Wilcoxon rank sum test, with correction for FDR. Adjusted *p* values deemed significant when adj *p*-value (P < 0.05) and trends (P < 0.10).

**Table 2:** Mean relative abundance (%) of the ten most abundant microbes, classified to the taxonomic level of family, in each dietary group.

Kingdom	Phylum	Class	Order	Family	WCPRG	PRG	*P*-value	adj *P*- value
Bacteria	Bacteroidetes	Bacteroidia	Bacteroidales	Prevotellaceae	43.90%	43.20%	0.86	0.94
Bacteria	Firmicutes	Clostridia	Clostridiales	Lachnospiraceae	13.00%	12.13%	0.32	0.51
Bacteria	Firmicutes	Clostridia	Clostridiales	Ruminococcaceae	11.23%	10.46%	0.25	0.51
Bacteria	Firmicutes	Clostridia	Clostridiales	Christensenellaceae	5.03%	5.24%	0.62	0.71
Bacteria	Bacteroidetes	Bacteroidia	Bacteroidales	Rikenellaceae	4.04%	4.33%	0.19	0.49
Bacteria	Fibrobacteres	Fibrobacteria	Fibrobacterales	Fibrobacteraceae	4.18%	3.93%	0.43	0.60
Bacteria	Firmicutes	Negativicutes	Selenomonadales	Veillonellaceae	2.40%	3.39%	<0.001	0.02
Bacteria	Bacteroidetes	Bacteroidia	Bacteroidales	F082	2.50%	2.35%	0.37	0.53
Bacteria	Firmicutes	Negativicutes	Selenomonadales	Acidaminococcaceae	1.69%	2.26%	0.01	0.18
Bacteria	Spirochaetes	Spirochaetia	Spirochaetales	Spirochaetaceae	1.63%	1.69%	0.93	0.97

PRG = Perennial ryegrass only; WCPRG = Perennial ryegrass and white clover sward;

*p*- values were generated using the Wilcoxon rank sum test, with correction for FDR. Adjusted *p* values deemed significant when adj *p*-value (P < 0.05) and trends (P < 0.10).

When classified at the genus level and corrected for FDR, the relative abundance of 15 taxa, with an average abundance of > 0.1% in at least one dietary group were found to be significantly, or tentatively, affected by diet (Table 3). Most genera influenced by diet belonged to the phylum *Firmicutes* (n = 11). A total of 4 genera belonging to the family *Lachnospiraceae* had relative abundances that differed between the groups. An increase in the relative abundance of*, Pseudobutyrivibrio* and *Lachnoclostridium 10* was evident in the PRG animals whereas there was a rise in the relative abundance of *Lachnospira* and *Lachnoclostridium 1* in the WCPRG group. Within *Veillonellaceae*, the abundance of *Selenomonas 1* was decreased in the animals grazing the WCPRG swards. In the family *Ruminococcaceae*, a near significant increase in the abundance of *Ruminococcaceae UCG-014* occurred in the WCPRG.

**Table 3:** Treatment associated differences classified to genus level with mean relative abundance (%) for each group.

Kingdom	Phylum	Class	Order	Family	Genus	WCPRG	PRG	*P*-value	adj *P*- value
Bacteria	Actinobacteria	Coriobacteriia	Coriobacteriales	Atopobiaceae	Olsenella	0.21%	0.15%	0.0022	0.032
Bacteria	Firmicutes	Clostridia	Clostridiales	Lachnospiraceae	Lachnospira	0.23%	0.04%	<0.001	<0.001
Bacteria	Firmicutes	Clostridia	Clostridiales	Lachnospiraceae	Pseudobutyrivibrio	0.81%	1.38%	<0.001	<0.001
Bacteria	Firmicutes	Clostridia	Clostridiales	Ruminococcaceae	Ruminococcus_2	0.16%	0.09%	<0.001	<0.01
Bacteria	Firmicutes	Clostridia	Clostridiales	Lachnospiraceae	Lachnoclostridium_1	0.13%	0.07%	<0.001	<0.01
Bacteria	Firmicutes	Negativicutes	Selenomonadales	Veillonellaceae	Selenomonas_1	2.05%	2.77%	<0.001	<0.01
Bacteria	Firmicutes	Erysipelotrichia	Erysipelotrichales	Erysipelotrichaceae	Kandleria	0.35%	0.16%	<0.001	0.015
Bacteria	Firmicutes	Negativicutes	Selenomonadales	Veillonellaceae	Selenomonas	0.05%	0.15%	<0.001	0.015
Bacteria	Firmicutes	Clostridia	Clostridiales	Lachnospiraceae	Lachnoclostridium_10	0.13%	0.19%	0.0016	0.025
Bacteria	Firmicutes	Clostridia	Clostridiales	Ruminococcaceae	Ruminococcaceae_UCG-014	1.35%	0.86%	0.0051	0.06
Bacteria	Firmicutes	Clostridia	Clostridiales	Ruminococcaceae	Ruminiclostridium_6	0.10%	0.07%	0.006	0.067
Bacteria	Firmicutes	Clostridia	Clostridiales	Ruminococcaceae	Saccharofermentans	1.58%	1.24%	0.007	0.072
Bacteria	Patescibacteria	Saccharimonadia	Saccharimonadales	Saccharimonadaceae	Candidatus_Saccharimonas	0.71%	0.51%	0.0034	0.045
Bacteria	Proteobacteria	Deltaproteobacteria	Desulfovibrionales	Desulfovibrionaceae	Desulfovibrio	0.07%	0.12%	0.0016	0.025
Archaea	Euryarchaeota	Methanobacteria	Methanobacteriales	Methanobacteriaceae	Methanobrevibacter	1.13%	1.54%	0.0046	0.058

PRG = Perennial ryegrass only; WCPRG = Perennial ryegrass and white clover sward.

*P*- values were generated using the Wilcoxon rank sum test, with correction for FDR. Adjusted *p* values deemed significant when adj *p*-value (P < 0.05) and trends (P < 0.10).

Only microbes with a relative abundance of > 0.1%, in one and/or both groups reported.

Diet also had a minor effect on the relative abundance of lowly abundant bacteria including an increase to the relative abundance of genera *Ruminococcus 2, Candidatus Saccharimonas*, *Kandleria* and *Olsenella*, in WCPRG cows and *Desulfovibrio* and *Selenomonas* in the PRG group. In addition, the relative abundance of *Methanobrevibacter* was increased in the PRG animals when abundance was calculated as a proportion of all 16S rRNA sequences.

### Effect of Sward Type on Rumen Archaeal Community

**Figure 2:**
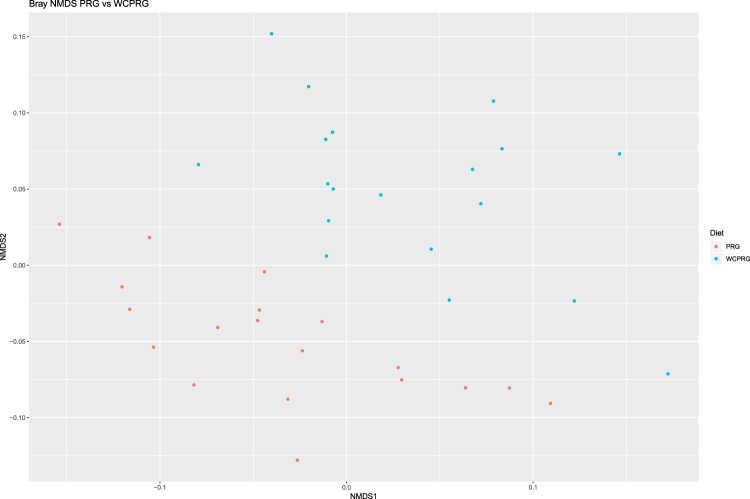
Bray-Curtis NMDS plot highlighting differences in the archaeal community composition between treatments. Different colour dots represent samples obtained from animals grazing different swards. Red = Perennial ryegrass (PRG), Blue = Perennial ryegrass and white clover (WCPRG).

Overall, the archaeal relative abundance accounted for a small proportion (1.68%) of all sequences generated. Within both dietary groups, *Methanobacteriaceae* dominated at the family level with the genera *Methanobrevibacter* accounting for 79.1% of archaea across all samples. Calculating the abundance of methanogens relative to archaeal sequences only, resulted in a degree of sample clustering based on diet (Fig. 2). In addition, an increase in the relative abundance of *Methanobrevibacter* (81.98 vs. 76.21%; adj P < 0.001) within the PRG diet with a subtle shift towards an increase in the abundance of *Methanosphaera* (10.19 vs. 15.40%; adj P < 0.001) in the WCPRG group, was observed. No dietary effect was observed on the remaining genera of methanogens examined in the current study.

### Predicted Metabolic Pathway Analysis

**Figure 3:**
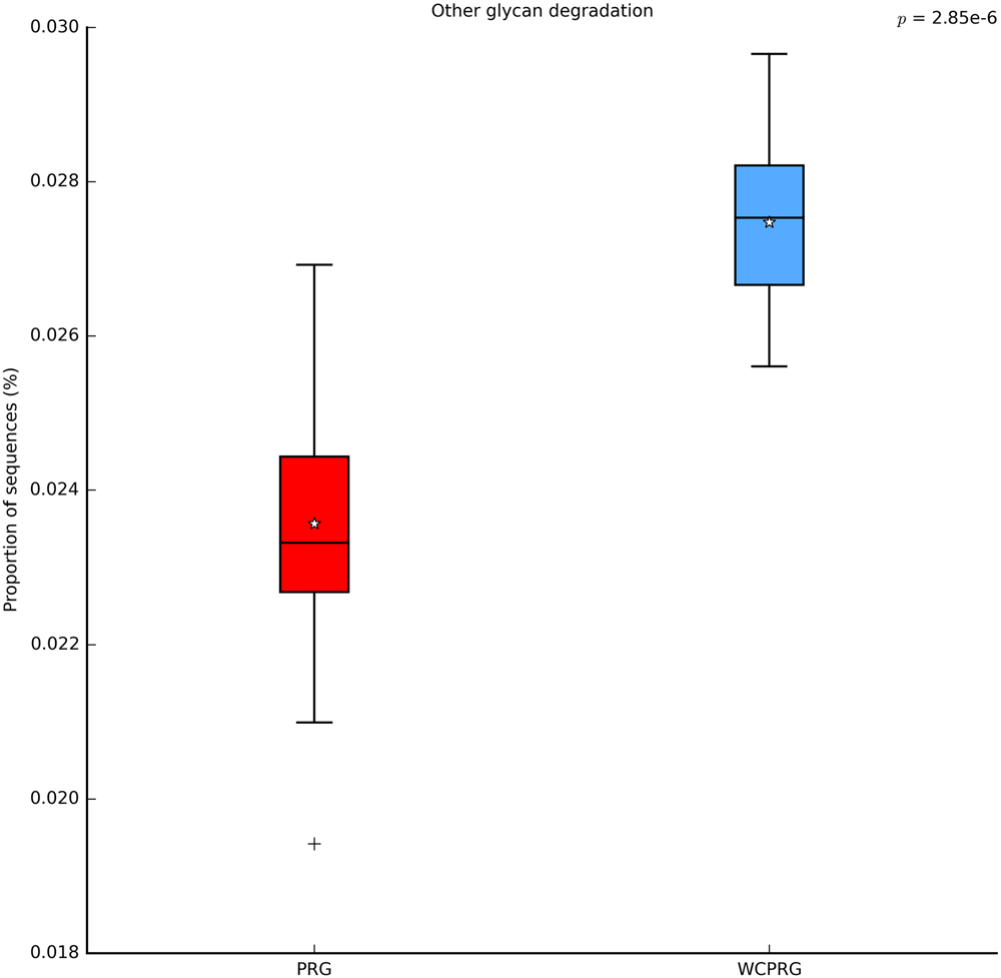
Relative read abundance for pathways predicted to be associated with *other glycan degradation*. PRG = Perennial ryegrass, WCPRG = Perennial ryegrass and white clover.

**Figure 4:**
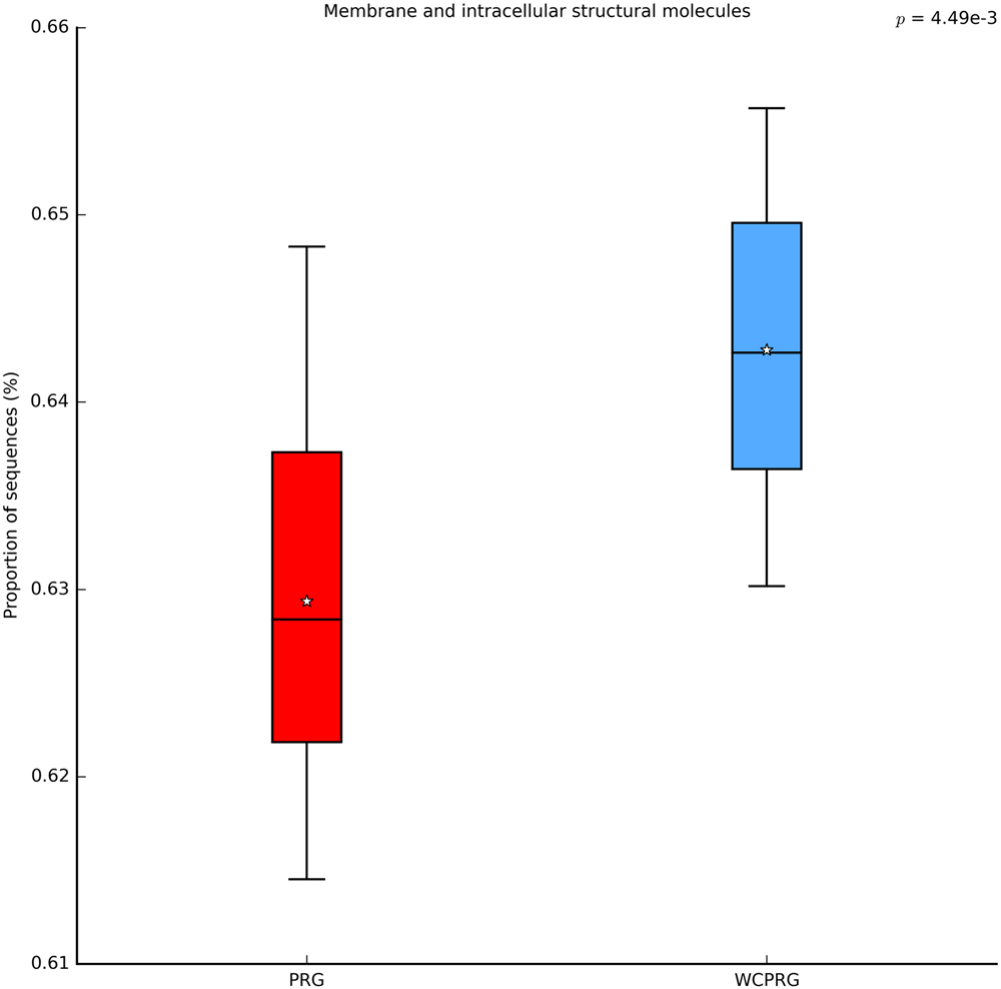
Relative read abundance for pathways predicted to be associated with *membrane and intracellular structural molecules*. PRG = Perennial ryegrass, WCPRG = Perennial ryegrass and white clover.

Two metabolic pathways, with a relative read abundance greater than 0.01%, were found to be influenced by diet. Metabolic pathways associated with *other glycan degradation* (adj P < 0.001; Fig. 3) and *membrane and intracellular structural molecules* (adj P < 0.01; Fig. 4) were predicted to have a higher expression in rumen samples collected from WCPRG cows. Diet did not have an impact on pathways predicted to be connected to *methane metabolism*.

## Discussion

The addition of white clover to the grazing sward has been shown to boost herbage yield and reduce the dependence on synthetic fertiliser^3–5^. Equally, animal performance has been shown to potentially benefit from an enhanced dietary nutritive value and digestibility with the incorporation of white clover into the grazing platform^6–10^, albeit response can be varied^11,12^.

The grazing of clover monocultures has previously been suggested to illicit stresses, such as low pH and/or bloat, on the rumen in conjunction with an elevated relative abundance of genera belonging to the phylum *Proteobacteria*^27^. However, data generated from this study would suggest the grazing of swards with 24% clover content to be free of imbalances to the rumen microbiota as the relative abundance of *Proteobacteria* between diets was consistent.

White clover has previously been shown to have higher pectin content in comparison to perennial ryegrass^15,16^. Therefore, elevated expression of pathways predicted to be associated with *other glycan degradation* in the WCPRG may be indicative of an increased supply of pectin in the diet. Members of the genera *Lachnospira*, such as *L. multiparus*, are predominantly pectin degrading bacteria^32^. Therefore, the observed greater abundance of this genus in the WCPRG cows is most likely due to higher pectin content in white clover when compared to grasses. This is similar to the findings of previous studies focused on legume forages^27,33^.

The ability to degrade pectin has been identified in other ruminal bacteria such as *Prevotella* and *Butyrivibrio*^34^. The genus *Prevotella* has been identified as a core rumen microbial resident^21^. In addition, the genus has been labelled a catabolic generalist^35^ due the ability of individual member species to degrade a variety of substrates such as starch, hemicellulose, xylan and pectin^36,37^. As a result, the dominance of *Prevotella* within both dietary groups is most likely reflective of the numerous growth substrates utilised by members of the genera. *Butyrivibrio* also has the ability to degrade a similar cohort of plant structural carbohydrates^38^, most likely leading to the similar relative abundance of the bacteria across both dietary groups.

The genomes of *Lachnospira, Prevotella* and *Butyrivibrio* have recently been reported to encode for pectin methyl esterases (PME)^39^, which are responsible for the conversion of pectin to methanol^40^. The abundance of methylotrophic methanogens such as *Methanosphaera*, is suggested to be limited by the availability of methanol in the rumen^40^. Therefore, the minor shift towards an increased abundance of *Methanosphaera* relative to the overall archaeal community, in the WCPRG dietary group, may be due to an enhanced supply of methyl compounds arising from the fermentation of pectin by the previously mentioned bacteria.

White clover is more readily fermented in the rumen in comparison to grasses^41^ and has been shown to be both associated with reduced ruminal retention time^42^ and increased feed intake^4,20,29^. Such characteristics of clover degradation may be a combination of the reduced fibre content in clover^14^ and cellular fracture, rather than indentation, of clover plant cells during the fermentation process^18^.

The model proposed by Janssen^43^ suggests that a faster rumen passage rate is likely to negatively impact microbial growth rates when substrates for growth are reduced. Recent work by others has identified *Pseudobutyrivibrio* to be a secondary coloniser of ingested fibre^44,45^, and therefore most likely unsuited to conditions associated with increased ruminal passage rate. In addition members of the genera *Pseudobutyrivibrio* are known fermenters of hemicellulose^38^. While in depth chemical analysis was not performed on the swards under investigation in this study^12^, perennial ryegrass has been shown to have a greater proportion of hemicellulose in comparison to white clover^16,17^. Therefore, the increase in the relative abundance of *Pseudobutyrivibrio* observed in the grass only group may have potentially benefited from an increased availability of hemicellulose. Also it is possible that clover may have some ‘as of yet’ unknown inhibitory effect on the microbe as a reduction in the abundance of the genus *in vitro* has previously been shown when incubated on red clover in comparison to PRG^46^.

Key characteristics of mixed clover and grass swards are indicative of their potential as a possible mitigation strategy for pastoral based systems. For example, a decreased rumen retention time has previously been associated with reductions in methane yield^47,48^ while reducing the proportion of fibre in the diet is also related to lower methane output^49^. We observed a minor reduction in the relative abundance of *Methanobrevibacter* in the clover sward when microbial abundance was calculated relative to all 16S rRNA sequences. However, as qPCR was not conducted as part of this study it is difficult to determine if the actual abundance of *Methanobrevibacter* was reduced. Equally, while the utilisation of 16S rRNA methods allowed for the simultaneous study of bacterial and archaeal populations in this study, the *mcrA* gene has proved a reliable biomarker for investigations of methanogenesis^50^. As a result, comparing the abundance of methanogens relative to bacteria on the bases of 16S rRNA sequencing, when studying methanogenesis, has limitations, including a lack of data on methanogen activity^51^. However, differences in the abundance of individual members of the methanogen population are known to be associated with methane production^52^. With this said when the abundance of methanogens was calculated relative to the archaeal sequences only, a subtle shift towards an increased abundance *Methanosphaera* was noted in WCPRG diet. In addition to this, a clear separation in the archaeal community structure between both dietary groups was apparent.

Theoretically, hydrogenotrophic methanogenesis has a greater net energy availability (−131 ΔG°′ (kJ/mol CH_4_))^53^ in comparison to methylotrophic methanogenesis (−113 to −112.5 ΔG°′ (kJ/mol CH_4_))^54^. However, as alluded to by Kelly *et al*.^40^, the lower H_2_ requirement of methylotrophic methanogenesis results in increased free energy availability in comparison to the CO_2_ reduction pathway. Previously, we referenced methanol to be the limiting factor for growth of *Methanosphaera* rather than H_2_. As a result, it could be possible that the increased supply of methanol, coupled with both the suggested negative effect of feed intake on members of *Methanobrevibacter*^55^ and more energetically favourable methanogenesis pathway, sustained a competitive advantage for *Methanosphaera* in the WCPRG group. While differences in the proportions of members of the archaeal community between both diets have been previously associated with reduced methane yield^56,57^, community shifts were minor which is reflective of the similar level of daily methane output and slight reduction in methane yield associated with the WCPRG diet.

Our research has shown the inclusion of white clover to the grazing sward to be accompanied by subtle changes to the composition of the rumen microbiome. Results from this study highlight the effect of different sward types on the composition of the rumen. It is assumed that more abundant microbes in a normal functioning rumen prosper due to their fermentation pathways providing an advantage, over less abundant microbial inhabitants, to utilise available substrates^43^. Considering this, it is probable that variation in the substrates supplied by the different swards and/or variation in ruminal retention time altered conditions in the rumen between the two groups. However, the subtle changes in nutrient flow to the rumen, do not appear to have negative consequences on the microbial composition indicative of ruminal stress, such as low pH or bloat, when grazing of swards with a 24% clover. To achieve benefits to animal performance, a 30% white clover inclusion rate is deemed necessary^58^. Therefore, future research may look to investigate if white clover, at an inclusion rate of 30% or greater, has similar impacts on the rumen microbiome.

## Methods

Animal handling and sampling procedures carried out in this experiment were recommended for licencing by two qualified signatories at University College Dublin and approved for licencing by the Irish Department of Health and Children in accordance with the legislative requirements under section 8 of the Cruelty to Animals Act, 1876.

The experiment described here is based on an earlier study which examined the effect of sward composition on milk production and enteric methane emissions of dairy cows grazing either perennial ryegrass dominant pasture with or without the inclusion of white clover. A more thorough description of the animal model, methane measurement methodology and sward characteristics is reported in the original study^12^.

### Animal and Grazing Model, DMI and Enteric Methane Emissions Estimates

Briefly, a total of 40 spring calving late lactation dairy cows, balanced by breed (Holstein Friesian, Norwegian Red and Norwegian Red × Holstein Friesian) were selected in April 2011 from the main herd at the Dairygold Research Farm (Teagasc, Animal and Grassland Research and Innovation Centre, Moorepark, Fermoy, Co. Cork, Ireland). Cows were randomly assigned to two herds and rotationally grazed swards containing either perennial ryegrass (*Lolium perenne*) only (PRG; n = 20) or perennial ryegrass and white clover (*Trifolium repen*s) (WCPRG; n = 20). A postgrazing sward height (PostGSH) of 4 cm above ground height was targeted to allow individual animals a daily herbage allowance (HA) of 16 kg of DM. Additionally, cows were allocated a supplementation of 1 kg of concentrate/cow per day (CP = 154.1, NDF = 40.9, and ash = 102.8 g/kg of DM). Concentrate supplementation was withdrawn three days prior to the measurement of dry matter intake (DMI), followed by an increase in HA to 17 kg of DM/cow per day.

The PRG swards contained a 50:50 mixture of tetraploid (AstonEnergy) and diploid (Tyrella) PRG varieties sown at 37 kg/ha. The WCPRG sward included the same relative PRG combination utilised in the PRG sward (37 kg/ha) in addition to a medium leaf white clover mixture of 50:50 (Chieftain and Crusader) sown at a rate of 5 kg/ha.

### Sample Collection and DNA Extraction

During the methane measurement period, approximately 50 ml of rumen digesta was sampled from each animal using transoesophageal rumen sampling (FLORA rumen scoop; Guelph, Ontario, Canada). After collection, individual rumen samples were immediately squeezed through three layers of synthetic cheesecloth and snap frozen followed by storage at −20 °C and by subsequent long-term storage at −80 °C until DNA was extracted. Further details in relation to the processing of samples at collection are reported in Enriquez-Hidalgo *et al*.^59^.

One sample was mislaid resulting in a total number of 39 samples (PRG n = 20; WCPRG n = 19) for microbial DNA extraction. Using the repeated bead beating and column purification method^31^, DNA was extracted from approximately 250 mg of the frozen rumen sample. DNA quality was assessed on agarose gels (see Supplementary Fig. S1) with the concentration of extracted DNA quantified on the Nanodrop 1000 spectrophotometer.

### Library Preparation and Sequencing

A total of 39 amplicon libraries were generated from 25 ng of individual rumen microbial DNA from a total of 39 animals by performing two rounds of PCR amplification as outlined in the Illumina Miseq *16S Sample Preparation Guide* with minor modifications to cycle length, as outlined by McGovern *et al*.^60^. An additional library was generated as an internal positive control using the ZymoBIOMICS^TM^ Microbial Community Standard DNA (DS) (Zymo Research Corp., Irvine, CA, United States) to assess library preparation and sequencing performance. The first round of PCR amplification, targeting the V4 hypervariable region of the 16S rRNA gene, was performed using the 515 F/806 R primers^61^, designed with Nextera over hang adapters, and 2x KAPA Hifi HotStart ReadyMix DNA polymerase (Roche Diagnositics, West Sussex, UK). Cycle conditions were as follows: 95 °C for 3 minutes, 20 cycles at 95 °C for 30 seconds, 55 °C for 30 seconds, 72 °C for 30 seconds and then 72 °C for 5 minutes.

Amplicons were purified using the QIAquick PCR Purification Kit (Qiagen, Manchester, UK). Following purification, amplicons were subject to a second round of PCR to permit attachment of dual indices and Illumina sequencing adapters using the Nextera XT indexing kit (Illumina, San Diego, CA, USA). Cycle conditions for the second round of PCR were 95 °C for 3 minutes, 8 cycles at 95 °C for 30 seconds, 55 °C for 30 seconds, 72 °C for 30 seconds and then 72 °C for 5 minutes followed by an additional PCR purification with the QIAquick PCR Purification Kit (Qiagen, Manchester, UK). Confirmation of amplicon generation was conducted visually on a 2% agarose gel. Amplicons were pooled together in equal concentration and subject to gel purification using the QIAquick Gel Extraction Kit (Qiagen, Manchester, UK) to remove adapter primers and further purified to remove any residues of agarose using the QIAquick PCR purification kit (Qiagen, Manchester, UK).

Pooled sample purity and quantity was analysed on the Nanodrop 1000 with further validation on the Qubit fluorometer and using the KAPA SYBR FAST universal kit with Illumina Primer Premix (Roche Diagnositics, West Sussex, UK). Following this, the library pool was diluted and denatured as per the Illumina Miseq *16S Sample Preparation Guide* with sequencing conducted on the Illumina MiSeq using the 500 cycle version 2 MiSeq reagent kit (Illumina, San Diego, CA, USA).

### Sequencing Analysis

All 16S rRNA gene amplicons were processed in *R* (version 3.4.2) using *DADA2* (version 1.9.0) (https://benjjneb.github.io/dada2/tutorial.html) and submitted to the pipeline as described^62^ with minor modifications. Quality checks of both forward and reverse reads were initiated based on a visualisation of the average Q score for each sample with the aim to ensure mean Q scores of > 30 were upheld for forward and reverse reads. To achieve this, forward reads were trimmed to a length of 240 bp and reverse reads trimmed to 200 bp. The removal of primer sequences was conducted using the trimLeft function. Identical sequences were combined using the dereplication function followed by the merging of forward and reverse reads. Following this an amplicon sequence variant (ASV) table was constructed after which chimeric sequences were removed and taxonomy assigned to sequences variants using the SILVA database (version 132). Sample metadata, sequence taxonomy, and ASVs were combined into a phyloseq object using *phyloseq* (version 1.22.3)^63^ for further analysis. Predictions of metabolic pathways for each sample, based on the generated ASVs, were conducted using CowPI^64^.

### Statistical Analysis

Analysis of animal performance data has been previously reported by Enriquez-Hidalgo *et al*.^12^. The generated ASV table and sequence taxonomies were analysed in *R* (version 3.4.2). Of the 39 samples sequenced, one sample was excluded from the analysis due to having a substantially lower sequencing depth compared to all other samples leaving a total of 38 successfully sequenced samples evenly split between the dietary groups (n = 19 per group). Taxa with a read count of less than two were removed for statistical analysis. The relative abundance of taxa was calculated for each sample at the genus level, as a percentage of total read count, in *phyloseq*. Two non-metric multidimensional scaling (NMDS) plots, based on Bray-Curtis dissimilarity were generated to visualise differences in the overall microbial communities (Fig. 1) and archaeal community (Fig. 2) between the dietary treatments. A PERMANOVA test, based on 9,999 permutations and a significance level of P < 0.05, was carried using the R package *vegan* (version 2.5.4)^65^ implemented through *microbiome* (version 1.0.2)^66^ to investigate differences in the overall community structure amongst samples, based on treatment, at the level of genus. The Wilcoxon rank sum test, with Benjamini Hochberg (BH) correction for false discovery rate (FDR) was implemented for the identification of significant treatment differences in the overall relative abundance of taxa and the archaeal only proportion, based on adjusted *P*-values (adj P) of <0.05. Only taxa with a relative abundance of >0.1% in at least one of the dietary groups, was considered in treatment comparisons. The dietary grouping of animals was the only descriptive variable included in the comparison of microbial communities between treatments, due to the balanced statistical design of the original study^12^ and dietary focus of the current study. A Student’s T-Test was used to compare diversity metrics microbial community (species level) structure comparisons. Statistical analysis of predicted pathways was carried out using STAMP (version 2.1.3)^67^. Comparison of predicted pathways was conducted using the proportion of reads that were annotated to each individual metabolic pathway as a percentage of the total reads (read relative abundance). Statistical analysis of read relative abundance was conducted using a Student’s T-Test, with BH correction for FDR (adj P < 0.05) to determine differences based on dietary group. Spearman’s rank correlation coefficient was used to determine the correlation of the theoretical composition of the ZymoBIOMICS^TM^ DS standard and that of over generated DS library.

## Supplementary information

Supplementary Fig. 1.

## Data Availability

16S rRNA gene amplicon sequence data for this experiment is accessible at the National Centre for Biotechnology Information Sequence Read Archive (SRA; http://www.ncbi.nlm.nih.gov/sra) accession number PRJNA550258.
