# Supplementary material for: Sward type alters the relative abundance of members of the rumen microbial ecosystem in dairy cows
Source: Sci Rep. 2020 Jun 9;10:9317. doi: 10.1038/s41598-020-66028-3 (PMC7283238; doi:10.1038/s41598-020-66028-3)
Supplement: Supplementary file 1 — Supplementary Fig. 1. [file 41598_2020_66028_MOESM1_ESM.pdf]

Supplementary Material

Sward type alters the relative abundance of members of the rumen microbial ecosystem in dairy cows

Authors: Paul E. Smith[1, 2], Daniel Enriquez-Hidalgo[3, 4, 5], Deirdre Hennessy[3], Matthew S. McCabe[1] David A. Kenny[1, 2], Alan K. Kelly[2], Sinead M. Waters[1]

[1]Teagasc, Animal and Bioscience Research Department, Animal & Grassland Research and Innovation Centre, Teagasc, Grange, Dunsany, Ireland
[2]UCD College of Health and Agricultural Sciences, University College Dublin, Belfield, Ireland
[3]Teagasc, Animal & Grassland Research and Innovation Centre, Moorepark, Fermoy, County Cork, Ireland
[4]University of Bristol, Bristol Veterinary School, Langford, Somerset, BS40 5DU, UK
[5]Departamento de Ciencias Animales, Facultad de Agronomía e Ingeniería Forestal, Pontificia Universidad Católica de Chile, Santiago, Chile

Correspondence:

Prof Sinead M. Waters

***sinead.waters@teagasc.ie**

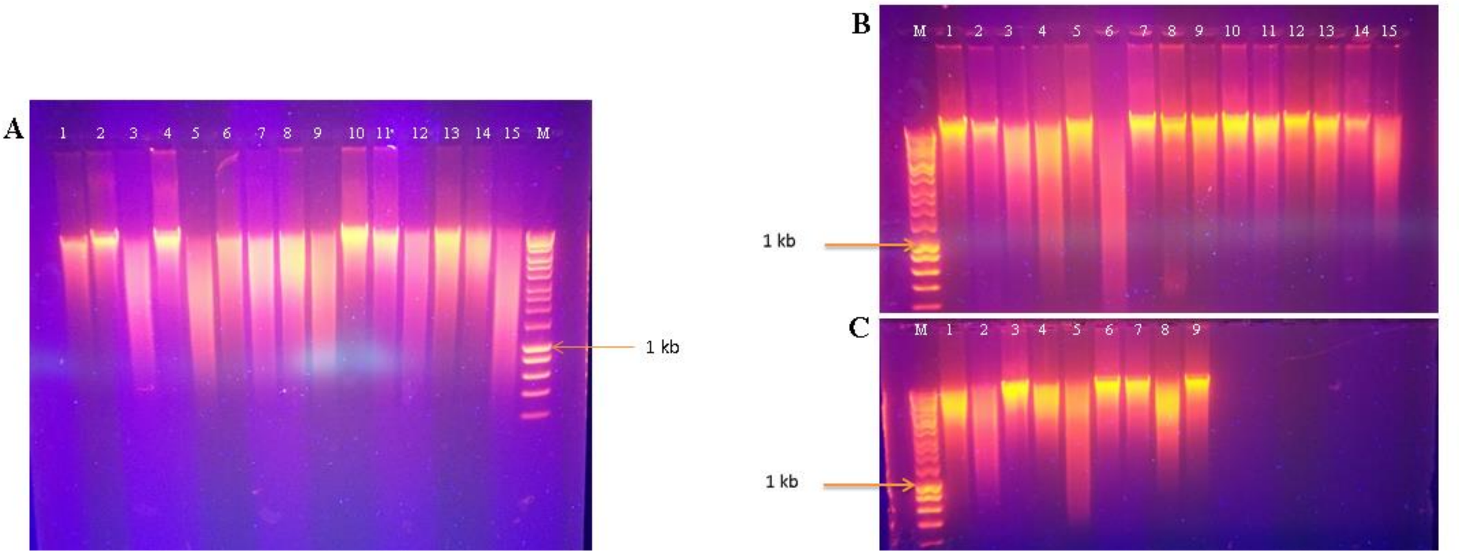

Supplementary Fig. 1 Quality of genomic DNA extracted from rumen samples visually assessed on a 0.8% agarose gel. Orange arrow indicates the position of the 1kb band as per the HyperLadder™ 1kb molecular weight marker (Bioline, London, UK). (A-C): rows of sample lanes; (1-15): individual sample lanes; (M) molecular weight. Samples in rows B and C were run on the same gel but have been separated by a white line for visualisation purposes.
